# Co-Location of QTL for Vigor and Resistance to Three Diseases in *Juglans microcarpa* × *J. regia* Rootstocks

**DOI:** 10.3390/ijms26030903

**Published:** 2025-01-22

**Authors:** Houston J. Saxe, Charles A. Leslie, Patrick J. Brown, Andreas Westphal, Daniel A. Kluepfel, Gregory T. Browne, Abhaya M. Dandekar

**Affiliations:** 1 Department of Plant Sciences, University of California, Davis, CA 95616, USA; hsaxe@ucdavis.edu (H.J.S.); caleslie@ucdavis.edu (C.A.L.); pjbrown@ucdavis.edu (P.J.B.); 2 Department of Nematology, University of California, Riverside, CA 92521, USA; andreas.westphal@ucr.edu; 3 USDA-ARS Crops Pathology and Genetics Research Unit, Department of Plant Pathology, University of California, Davis, CA 95616, USA; daniel.kluepfel@usda.gov (D.A.K.); gregory.browne@usda.gov (G.T.B.)

**Keywords:** walnut, QTL, host–pathogen interaction, rootstock, genomic selection, marker-assisted selection

## Abstract

A QTL on chromosome 4D of the *Juglans microcarpa* × *J. regia* genome that co-located resistance against *Agrobacterium tumefaciens*, *Phytophthora pini,* and *Phytophthora cinnamomi* disease scores was investigated for additional traits. Phenotypic data for *Pratylenchus vulnus* counts and tree height were analyzed in this study for the same hybrids previously used to identify this QTL. Using the same GBS genotype data, the same co-located QTL for *A. tumefaciens* and *Phytophthora* spp. disease scores were reproduced and the QTL for *P. vulnus* counts and tree height were co-located with resistance to *A. tumefaciens* and *Phytophthora* spp. Moreover, we found GBS genotype data to harbor additional genetic variation unrelated to any of the traits analyzed. Marker-assisted and genomic selection models were created and assessed for their performance in selection. The ability to predict traits using SNP data was strongest with two-year tree height, followed by *A. tumefaciens* disease score, three-year tree height, *Phytophthora* spp. disease score, and *P. vulnus* counts. These results suggest a shared mechanism of action that links disease to tree height. Moreover, deploying these selection models would assist efforts in walnut improvement for rootstock genotypes.

## 1. Introduction

Walnuts make a substantial contribution to the California economy, ranking 10th among all California commodities and valued at USD 1.022 billion in 2021 [1]. Crown gall (caused by *Agrobacterium tumefaciens*), *Phytophthora* crown and root rot (*Phytophthora* spp.), and root lesion nematode (*Pratylenchus vulnus*) are important rootstock diseases of California walnuts that reduce their value. *Agrobacterium tumefaciens* is a rod-shaped, Gram-negative soil bacterium, while *Phytophthora* spp. are soilborne oomycetes. These microbes obstruct the vascular tissues with tumors (*A. tumefaciens*) or kill root, crown, and trunk tissues directly (*Phytophthora* spp.). Both pathogens cause disease by inhibiting the flow of nutrients and water to the grafted scion, the upper portion of the plant, reducing crop productivity while imperiling plant health. *Pratylenchus vulnus*, an obligate migratory endoparasitic nematode, causes crop losses by damaging the root cortical tissue and consuming plant nutrients, which ultimately impedes the flow of nutrients and water to the scion [2]. The most recent estimate suggests that these root diseases collectively cost the California walnut industry USD 241 million (~20% of the total farm gate value) per year [3].

Quantitative Trait Locus (QTL) analysis provides a powerful approach to locate and understand the functional basis of important genetic traits in tree crops. QTL mapping studies in tree crops have identified numerous QTL for various growth-related traits, such as stem diameter, tree height, and the number of whorls, providing valuable insights into the genetic control of these traits. For example, a study on *Hevea brasiliensis* identified 24 QTL for stem diameter, 7 for tree height, and 7 for the number of whorls [4]. Similarly, a study on *Populus deltoides* and *Populus simonii* hybrids identified a total of 208 QTL for drought-related traits, leading to the development of molecular identification markers for drought tolerance [5]. Most relevantly, co-located QTL for resistance to *Agrobacterium tumefaciens* disease score, *Phytophthora pini*, and *Phytophthora cinnamomi* were found on chromosome 4D of the *Juglans microcarpa* × *Juglans* regia genome [6]. It is important to note that most traits in tree crops are influenced by a large number of underlying QTL, and more research is needed to capture a large proportion of the genetic variation [7]. Therefore, QTL analysis provides valuable information for understanding the genetic basis of important traits in tree crops and has the potential to complement functional genomics analyses for crop improvement [4,5,6,7,8].

Another beneficial outcome of QTL analysis is the foundation for marker-assisted selection (MAS). MAS has demonstrated significant efficacy in tree crops compared to no selection, particularly in expediting the breeding process and enhancing the accuracy of trait selection. Once a QTL has been identified, these markers are used to predict phenotypes in breeding lines with unknown phenotypes to pre-select for potentially beneficial accessions. This contrasts with traditional methods, where selection is based solely on the often laborious determination of phenotypic traits. Several studies have highlighted the advantages of MAS, such as its ability to improve the efficiency of plant breeding, especially in tree species where breeding is time-consuming and challenging due to the genetic complexity of traits [9]. The use of MAS led to higher genetic gain and cost efficiency compared to traditional selection methods when dealing with complex traits controlled by multiple genes [10,11]. In grapes, marker-assisted selection increased the selection efficiency for seedlessness in the fruit. The author’s in silico analysis started with only 52% of the 1012 plants being seedless, and after selection 92% of the 547 plants were seedless [10]. While genomic selection has gained prominence, MAS remains a valuable tool with traits controlled by a few or single loci.

Use of QTL analysis in conjunction with other omics approaches has several advantages over the use of QTL analysis alone, which results in uneven sampling of the genome and potentially missing critical markers [12]. Combining QTL analysis or GWAS with transcriptomic, proteomic, or metabolomic approaches is becoming increasingly popular and shows promise in identifying potential causal genetic-trait relationships [13]. A major QTL had been found in walnut rootstocks for resistance to *A. tumefaciens* (crown gall) and *Phytophthora* spp. in *Juglans* hybrids created by crossing *J. microcarpa* half-sib mother trees DJUG 31.01 (henceforth 31.01) and DJUG 31.09 (henceforth 31.09) with the cultivated *J. regia* cv. Serr (henceforth cv. Serr) [6]. A small subset of the hybrids from the breeding populations used previously were analyzed in this study using transcriptomics. The differentially expressed genes suggested that cell wall biogenesis was involved in resistance to these pathogens and tree height [2]. In the work reported here, the QTL for *A. tumefaciens* and *Phytophthora* spp. disease scores, and a novel QTL for tree height and *P. vulnus* counts, all were co-located in the same region. Furthermore, these QTL were employed for genomic selection and marker-assisted selection with exceptional selection accuracies. This research builds on the foundation of molecular biology research in walnuts by providing further clues to root disease mechanisms and adds value to the breeding efforts in the industry by reducing the ratio of work inputs to desirable genetic output.

## 2. Results

### 2.1. QTL Mapping and Marker Selection

To discover markers for *P. vulnus* counts and tree height at two and three years of tree age, the mapped markers of 31.01, 31.09, and cv. ‘Serr’ from seedlings of 31.01 × ‘Serr’ and 31.09 × ‘Serr’ were downloaded from Ramasamy et al. [6] (Supplementary Table S2 of the paper). See methods Section 4.3, “Phenotypic Analysis”, for detailed definitions of the traits analyzed in this study. For 31.01, no markers were removed by the minor allele frequency (MAF) threshold of 0.1, 52 markers were removed by the missing sample of 0.95, no samples were removed by the missing marker threshold of 0.5, and 2152 data points (0.64% of the data) were imputed. For 31.09, 2 markers were removed by the MAF threshold of 0.1, 5 markers were removed by the missing sample of 0.95, no samples were removed by the missing marker threshold of 0.5, and 417 data points (0.11% of the data) were imputed. For cv. ‘Serr’ from the cross with 31.01, no markers were removed by the MAF threshold of 0.1, 40 markers were removed by the missing sample of 0.95, 1 sample was removed by the missing marker threshold of 0.5, and 1514 data points (0.56% of the data) were imputed. For cv. ‘Serr’ from cross with 31.09, no markers were removed by MAF threshold of 0.1, 42 markers were removed by missing sample of 0.95, no samples were removed by missing marker threshold of 0.5, and 1177 data points (0.41% of the data) were imputed.

For the 31.01 haplotype, recursive feature elimination showed that markers 31.01_Jm4D_26168643, 31.01_Jm4D_26359154, and 31.01_Jm4D_26669075 were shared amongst the most important eight predictors by mean decrease in impurity (MDI) across two- and three-year tree heights, and two- and three-year *P. vulnus* counts, *A. tumefaciens* disease score, and *Phytophthora* spp. disease score (File S1). For the 31.09 haplotype, recursive feature elimination showed that markers X31.09_Jm4D_26359154, X31.09_Jm4D_25625822, and X31.09_Jm4D_25101968 were shared amongst the most important eight predictors across two- and three-year tree heights, and three-year *P. vulnus* counts, A. *tumefaciens* disease score, and *Phytophthora* spp. disease score but not two-year *P. vulnus* counts (File S2). Overall, most of the important predictors were markers from chromosome Jm4D. rrBLUP coefficients of these markers for each trait showed that they were amongst the largest negative effects of the markers selected by the *rfe* function and indicated that disease resistance and lower tree height were associated with the b allele (Files S3 and S4, Figures S1 and S2).

**Figure 1:**
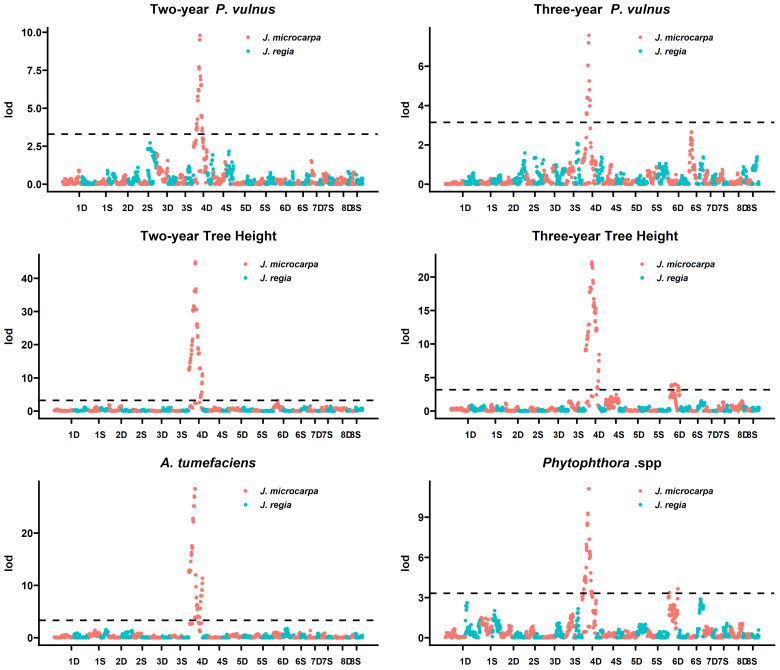
LOD plots for each of the six traits mapped to 31.01 and cv. Serr haplotypes. Chromosome numbers are on the x-axis, and LOD score is on the y-axis. The horizontal dashed line indicates the threshold above which LOD scores are significant.

**Figure 2:**
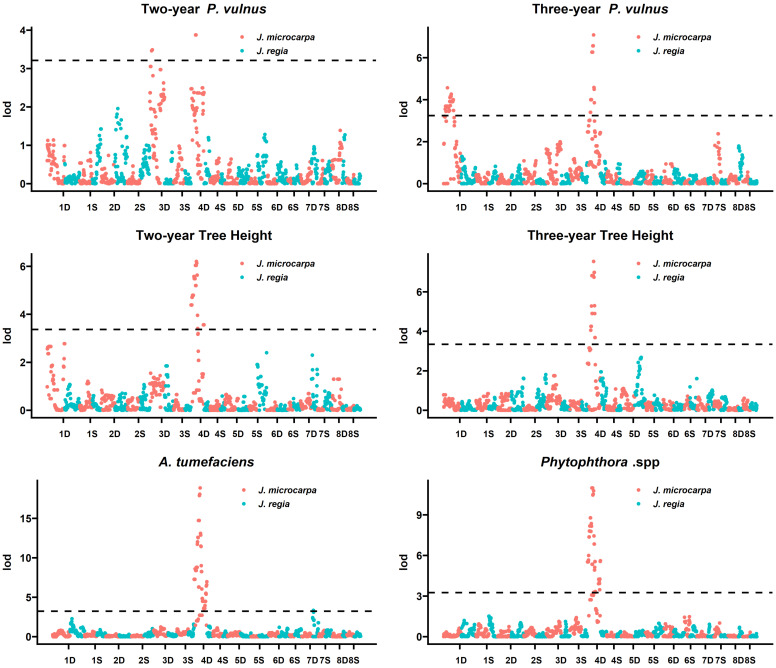
LOD plots for each of the six traits mapped to the 31.09 and cv. Serr haplotypes. Chromosome numbers are on the x-axis, and LOD score is on the y-axis. The horizontal dashed line indicates the threshold above which LOD scores are significant.

Moreover, logarithm of the odds (LOD) scores displayed major distinct peaks along 31.01 and 31.09 chromosomes 4D for each trait except for two-year *P. vulnus* counts in the 31.09 haplotype (Figure 1 and Figure 2). LOD significance thresholds at α = 0.05 for 31.01 × cv. Serr were 3.3, 3.1, 3.2, 3.2, 3.3, and 3.3 for two- and three-year *P. vulnus* counts, two- and three-year tree height, *A. tumefaciens* disease score, and *Phytophthora* spp. disease score, respectively. These thresholds resulted in 31, 17, 65, and 59 significant markers on Jm4D for two- and three-year *P. vulnus* counts, two-year tree height, and *A. tumefaciens* disease score, respectively (Table 1). The markers for three-year tree height and *Phytophthora* spp. disease score were split into 65 and 14, and 38 and 3 for chromosomes 4D and 6D, respectively. Furthermore, these markers spanned 12,109,447, 5,298,334, 30,660,994, and 30,060,179 base pairs on Jm4D for two- and three-year *P. vulnus* counts, two-year tree height, and *A. tumefaciens* disease score, respectively. The distances for three-year tree height and *Phytophthora* spp. disease score QTL were split into 30,660,994 and 3,493,793, and 21,190,695 and 3,720,218 base pairs for chromosomes 4D and 6D, respectively. The top markers by LOD score were 31.01_Jm4D_26669075 two- and three-year *P. vulnus* counts and two- and three-year tree height (Table 2). For *A. tumefaciens* and *Phytophthora* spp. disease scores, 31.01_Jm4D_26168643, 31.01_Jm4D_26359154, and 31.01_Jm4D_26669075 were shared as top makers because they had the same LOD and covered 500,432 base pairs on chromosome 4D.

For 31.01 × cv. Serr, these QTL explained 13.9%, 13.1%, 70.3%, and 45.4% of the variation in the phenotypic data for two- and three-year *P. vulnus* counts, two-year tree height, and *A. tumefaciens* disease score, respectively, all from Jm4D (Table 1). The variances explained for three-year tree height and *Phytophthora* spp. disease score were split into 41.4% and 4.8%, and 21.0% and 6.1% for chromosomes 4D and 6D, respectively. Conversely, just the top marker from each QTL explained 15.7%, 12.9%, 67.1%, 33.8%, 44.1%, and 18.9% for two- and three-year *P. vulnus* counts, two- and three-year tree height, *A. tumefaciens* disease score, and *Phytophthora* spp. disease score, respectively (Table 2).

LOD significance thresholds at α = 0.05 for 31.09 × cv. Serr were 3.2, 3.2, 3.4, 3.3, 3.2, and 3.3 for two- and three-year *P. vulnus* counts, two- and three-year tree height, *A. tumefaciens* disease score, and *Phytophthora* spp. disease score, respectively. These thresholds resulted in 31, 19, and 46 significant markers on Jm4D for two- and three-year tree height and *Phytophthora* spp. disease score, respectively (Table 3). For two-year *P. vulnus* counts, these markers were split into amounts of three and two for chromosomes Jm4D and Jm3D, respectively. For three-year *P. vulnus* counts, these markers were split into amounts of 14 and 39 for chromosomes Jm4D and Jm 1D, respectively. For *A. tumefaciens* disease score, these markers were split into amounts of 59 and 1 for chromosomes Jm4D and Jm7D, respectively.

These markers spanned 21,274,993, 10,480,961, and 29,549,284 bases on Jm4D for two- and three-year tree height, and *Phytophthora* spp. disease score, respectively (Table 3). For two-year *P. vulnus* counts, these distances were split into 54,916 and 372,371 base pairs for chromosomes Jm4D and Jm3D, respectively. For three-year *P. vulnus* counts, these distances were split into 9,378,085 and 24,916,299 base pairs for chromosomes Jm4D and Jm 1D, respectively. For *A. tumefaciens* disease score, these distances were split into 31,696,222 and 0 base pairs for chromosomes Jm4D and Jm7D, respectively.

The top marker(s) by LOD score were X31.09_Jm4D_23906200, X31.09_Jm4D_23960954, and X31.09_Jm4D_23961116 for two-year *P. vulnus* counts, X31.09_Jm4D_26359154 for three-year *P. vulnus* counts, 31.09_Jm4D_25101968 for two-year tree height, X31.09_Jm4D_26359154 for three-year tree height, X31.09_Jm4D_26359154 for *A. tumefaciens* disease score, and X31.09_Jm4D_23816262 and X31.09_Jm4D_24259264 for *Phytophthora* spp. disease score. Two-year *P. vulnus* counts had three top markers because the LOD scores were the same. These top markers spanned 54,916, 0, 0, 0, 0, and 443,002 base pairs for two- and three-year *P. vulnus* counts, two- and three-year tree height, *A. tumefaciens* disease score, and *Phytophthora* spp. disease score, respectively.

For 31.09 × cv. Serr, the variance explained in the phenotypic data for these QTL was 7.0% and 8.2% on chromosomes Jm3D and 4D for two-year *P. vulnus* counts, 8.5% and 16.7% on chromosomes Jm1D and 4D for three-year *P. vulnus* counts, 20.1% on chromosome Jm4D for two-year tree height, 17.2% on chromosome Jm4D for three-year tree height, 39.7% and 7% on chromosomes Jm4D and Jm7D for *A. tumefaciens* disease score, and 17.5% on chromosome Jm4D for *Phytophthora* spp. disease score (Table 3). Conversely, just the top marker from each QTL explained 8.2%, 17.3%, 23.5%, 17.1%, 36.9%, and 17.6% for two- and three-year *P. vulnus* counts, two- and three-year tree height, *A. tumefaciens* disease score, and *Phytophthora* spp. disease score, respectively (Table 4).

The cv. Serr haplotype showed no such QTL peaks nor did it have any significant markers for any trait in either cross (Figure 1 and Figure 2).

**Figure 3:**
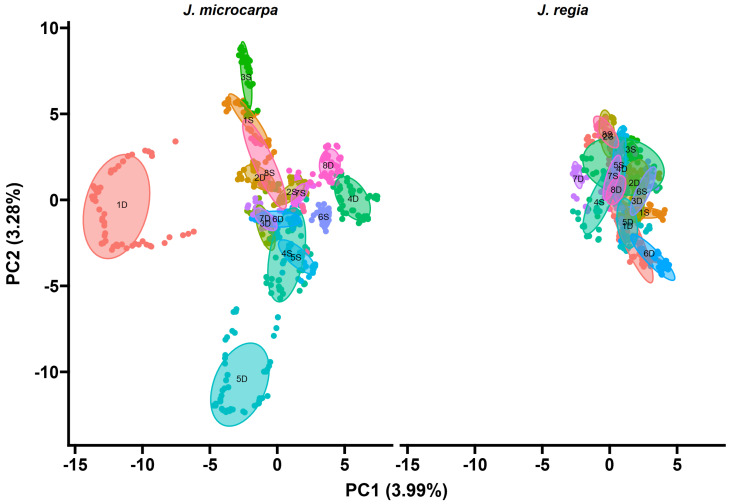
Scatter plot of Principal Component Analysis (PCA) loadings of all *Juglans microcarpa* 31.01 × cv. Serr markers discovered by GBS. The plot is facetted by parental haplotype. Each point represents a marker. All points and ellipses are colored and labeled by their chromosome of origin. Ellipses are labeled by chromosome of origin in black.

**Figure 4:**
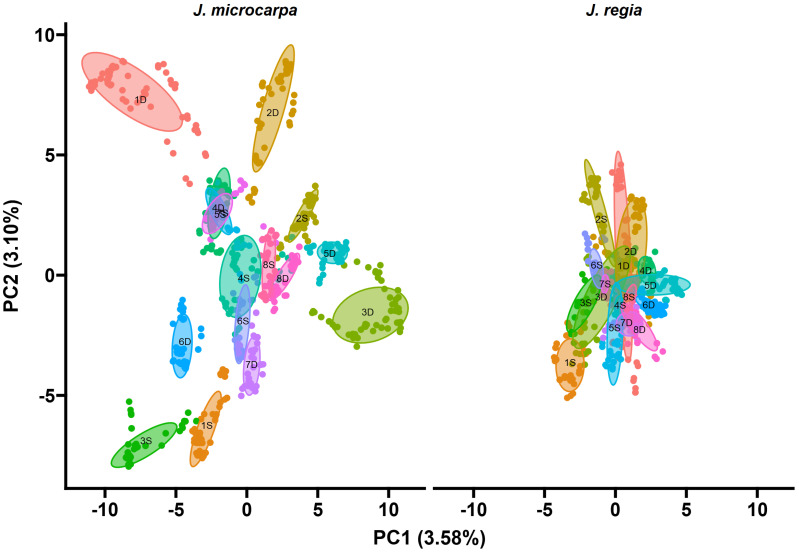
Scatter plot of Principal Component Analysis (PCA) loadings of all *Juglans microcarpa* 31.09 × cv. Serr markers discovered by GBS. The plot is facetted by parental haplotype. Each point represents a marker. All points and ellipses are colored and labeled by their chromosome of origin. Ellipses are labeled by chromosome of origin in black.

The top markers were highly correlated with each other, as were many other markers that tended to correlate by chromosome (Figure 3 and Figure 4). Moreover, PCA suggested that chromosome 4D is just one part of the total variation in the GBS data. In the 31.01 haplotype, chromosome 3S explained about as much variation in the data as 4D, and 5D and 1S explained about double the variation in the data as chromosome 4D (Figure 3). In the 31.09 haplotype, chromosome 4D explained a relatively minor amount of variation in the GBS data relative to the other chromosomes.

### 2.2. Prediction Accuracy of RF and rrBLUP Models to Discover Markers for P. vulnus

We used the *rfe* function from the caret package to estimate not which but how many markers to use in the marker-assisted selection (MAS) approach via random forest (RF) modeling. By default, the *rfe* function retained the top four markers for two-year tree height and two- and three-year *P. vulnus* counts and *A. tumefaciens* disease score. Conversely, for three-year tree height and *Phytophthora* spp. disease score, the *rfe* function retained all 946 markers. For our genomics selection (GS) approach, the ridge regression (rrBLUP) function uses all markers by default, albeit differently than standard regression.

To avoid model overfitting, we manually retained the top eight markers from the random forest function for three-year tree height and *Phytophthora* spp. disease score, which resulted in increased prediction accuracy over the default 946 markers selected. Using the top eight markers for modeling seemed to produce optimal prediction accuracies across all traits. Please see Methods Section 4.6. “Prediction and Selection Performance Estimation” for more details.

**Figure 5:**
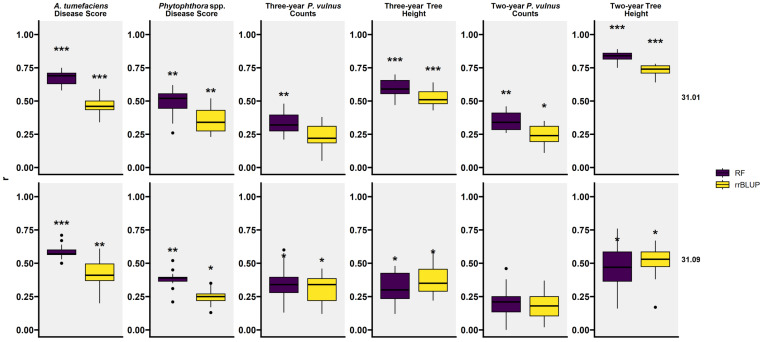
Prediction accuracy (r) and their significance for each trait assessed by Pearson correlation of model-predicted vs. actual values. There are four boxplots per trait representing two selection methods by two mother parents. The two methods tested were random forest (RF) from the caret package in R and functions from the rrBLUP (rrBLUP) package also from R. Stars indicate the average *p*-value for each boxplot of correlations. “***” average *p*-value less than 0.001, “**” average *p*-value between 0.01 and 0.001, “*” average *p*-value between 0.05 and 0.01.

The predictive accuracy of the top markers for each trait was assessed by a machine learning approach and correlation analyses using the randomForest and rrBLUP packages in R. RF and rrBLUP machine learning models resulted in the greatest prediction accuracy for two-year tree height followed by *A. tumefaciens* disease score, three-year tree height, *Phytophthora* spp. disease score, two-year *P. vulnus* counts, and three-year *P. vulnus* counts (Figure 5 and Table 5). On average, the RF approach outperformed rrBLUP, and predictions made in the 31.01 haplotype outperformed predictions made in the 31.09 haplotype. The only scenarios where the average *p*-value fell below the significance threshold of 0.05 were two and three-year *P. vulnus* counts and two-year tree height with the rrBLUP modeling method in the 31.09 maternal haplotype and three-year *P. vulnus* counts with the rrBLUP method in the 31.01 maternal haplotype.

**Figure 6:**
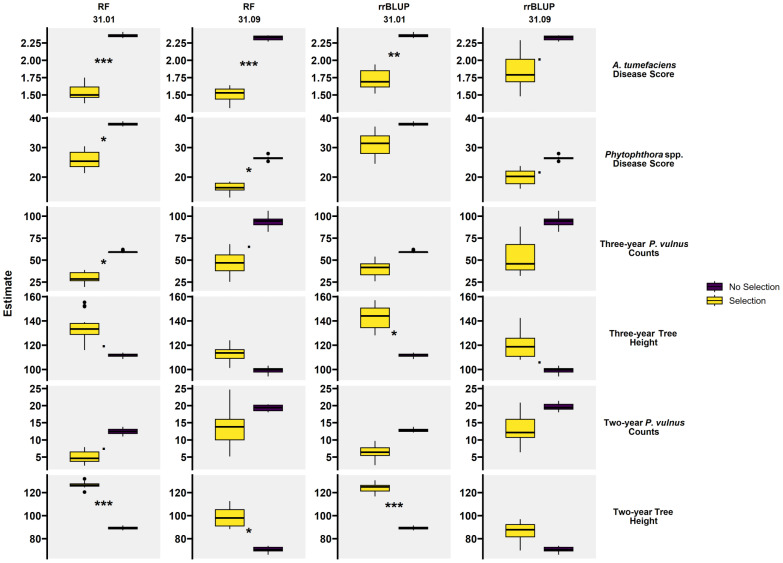
Selection performance by trait, method, and female parent. Boxplots represent phenotypic values (y-axis) of traits comparing *t*-test estimates of using selection vs. no selection. Stars indicate the average *p*-value for each boxplot of correlations. “***” average *p*-value less than 0.001, “**” average *p*-value between 0.01 and 0.001, “*” average *p*-value between 0.05 and 0.01, “.” Average *p*-value between 0.01 and 0.1. Dots above and below boxplots represent outliers.

To assess prediction accuracy more practically, the null hypothesis that artificial selection had no effect on phenotypic values was tested. To do this, we arranged each data split’s actual values by their predicted values, then selected the top 25% of the data and extracted only the actual values. These data became the “selected values”. We then compared the selected values to all the actual values (no selection) using a Student’s t-test. This was repeated 15 times for each trait and method combination. As with the performance assessment by correlation, on average, the RF approach outperformed rrBLUP, and predictions made in the 31.01 haplotype outperformed predictions made in the 31.09 haplotype (Figure 6 and Table 6). In all traits, the use of selection resulted in more desirable phenotypic values. However, average *p*-values fell below the significance threshold of 0.05 for two and three-year *P. vulnus* counts for all instances of the rrBLUP modeling method and the RF method in just the two-year *P. vulnus* counts-female parent 31.09 instance. Moreover, the rrBLUP modeling method’s average *p*-values fell below the significance threshold for all traits under the 31.09 haplotype. Other selection analyses falling below the significance threshold included all instances of the rrBLUP method for *Phytophthora* spp. disease score. The scenarios with the highest significance were two-year tree height in the 31.01 haplotype regardless of the modeling method and *A. tumefaciens* disease score with RF regardless of the maternal haplotype. Selection for *Phytophthora* spp. disease score yielded high levels of significance with the RF approach, while that of two- and three-year *P. vulnus* counts had lower values.

## 3. Discussion

### 3.1. Co-Located QTL

Machine learning methods were successfully implemented in QTL analyses [14,15] and using non-traditional and traditional statistical methods reproduced the co-located QTL spanning, 31.01_Jm4D_26168643, 31.01_Jm4D_26359154, and 31.01_Jm4D_26669075, for *A. tumefaciens* disease score and *Phytophthora* spp. disease score discovered by [6]. Moreover, the QTL for two- and three-year tree height and two- and three-year *P. vulnus* counts mapped to the same region (Figure 1 and Figure 2). While the co-located QTL were also present in the 31.09 × cv. Serr progeny, the peaks were not as high, and their exact location varied slightly between traits. This result raises some fundamental questions. Why would tree height also be under this QTL? Do any gene(s) within these QTL have SNPs? What are these gene(s) and their function(s)? The co-location of this many QTL for disease resistance suggests a nonspecific and robust disease resistance mechanism, as a gene-for-gene or induced mechanism seems unlikely for such broad-spectrum resistance. Moreover, the co-location of two- and three-year tree height QTL, traits seemingly unrelated to pathogenesis, to the same loci supports the hypothesis that the resistance is due to some pre-formed factor potentially related to the tree’s height. One such pre-formed factor is the plant cell wall, which is known to affect both the growth and disease-resistance characteristics of plants [6,16,17,18,19,20]. A recent study analyzing transcriptomes of four hybrids from this study and three from another breeding population found that gene expression coding for cell wall biogenesis was a significant biological process correlated with tree height, *A. tumefaciens* root and crown gall size, and *Phytophthora* spp. root and crown rot [2]. Saxe et al. found that increasing the expression of cell wall biogenetic genes was correlated with increasing tree height, and susceptibility to *A. tumefaciens* and *Phytophthora* spp. Similarly, we found that all traits co-located to this QTL on chromosome 4D showed the same relationship. Increasing tree height and increasing susceptibility to all pathogens was associated with the ‘a’ allele and decreasing tree height and resistance to all pathogens was associated with the ‘b’ allele (Files S3 and S4). Given the size of the LOD peaks for the QTL, fine mapping with higher marker density or a multi-omics approach has a higher chance of increasing the understanding of what genes or genetic elements are involved in the causality of these traits. Fine mapping could also improve the resolution of said QTL and may further increase the accuracy of selection models. In addition to fine mapping using higher marker densities, one could also employ transcriptomics to reveal the impact of the SNPs on the functional part of the genome. As has been observed in most RNAseq studies, not just one gene is differentially expressed, but thousands are [2]. And these genes can be analyzed for enrichment in biological processes correlated with the trait, giving more than just gene-level insights.

### 3.2. MAS and GS Prediction Accuracy

While QTL analysis can help understand the genetic basis underlying phenotypes, it also offers the potential for high throughput selection. Co-located QTL have been reported in these *J. microcarpa* × *J. regia* hybrids [6]; however, their ability to predict phenotypes was not assessed. We found a wide range in the ability of the selected markers to predict these phenotypes. Given that the predictors for each trait were largely the same, it seems likely that there are varying degrees of environmental or technical variation skewing the phenotypic data. For example, tree height, *A. tumefaciens* disease score (root and crown gall size), and *Phytophthora* spp. disease score (root and crown rot) are direct measures of the plant or plant response. Conversely, *P. vulnus* counts were generated by counting the number of nematodes per gram of root, an indirect measure of the host response. Perhaps the relationship between root lesions caused by *P. vulnus* and these QTL would be greater than the nematode count. Another reason for the large differences in prediction accuracy could be the genomic sequencing approach. Genotyping-by-sequencing was used to generate SNPs for this population. While this method is a powerful tool for SNP discovery, it has the drawback of potentially sampling the genome unevenly [12], thus missing critical markers for some traits. We observed that predictions using a MAS approach with fewer markers were better than the those found with the GS approach, which used all available markers with regularization (Figure 5 and Figure 6, Table 5 and Table 6). This is likely due to the presence of such a major QTL in each cross as MAS tends to outperform GS in such cases, while complex traits regulated by many QTL favors the use of GS [21,22].

While the predictive models have varying accuracies, they can be deployed in the same breeding population from which they were built to increase the efficiency of the artificial selection of the traits analyzed in this study. For example, utilizing these models to enhance rootstock selection for *A. tumefaciens* disease score and tree height would be the most effective given that they yielded the highest accuracies (Figure 5 and Table 5), and that their simulated selection effectiveness was the most significant (Figure 6 and Table 6). While two-year tree height yielded the highest accuracy overall, the three-year height data were more relevant, realistic, and prudent, as the prediction accuracy decreased substantially from two years to three years. For example, relying on a model based on the two-year data may overestimate the true accuracy of the model, as these trees develop and live much longer than two years.

## 4. Materials and Methods

### 4.1. Plant Material

“*Juglans microcarpa* trees DJUG 31.01 and DJUG 31.09 (USDA National Clonal Germplasm Repository, Davis, https://www.ars.usda.gov/pacific-west-area/davis-ca/natl-clonal-germplasm-rep-tree-fruit-nut-crops-grapes/, accessed on 10 January 2020) were used as mother trees in hybridization with *J. regia* cv. Serr in the springs of 2012–2015. Female flowers were sealed in pollen-impermeable semi-porous bags (PBS 10-1, PBS International, Crawley, UK) prior to opening, using non-absorbent cotton (Custom Hospital, Clackamas, OR, USA) to cushion and seal the bags around the branch after male flowers had been removed. When female flowers began opening (at the stigma separation stage), previously collected cv. Serr pollen was injected into the bags using a syringe. The bags were removed 3–4 weeks after pollination and immature nuts were tagged. The nuts were collected while still immature and with intact hulls (July–August), and stored refrigerated (up to several weeks). Embryos were extracted from nuts, germinated in vitro, and micropropagated to produce clones for disease resistance testing” [6].

### 4.2. GBS and SNP Discovery

“Genomic DNA was isolated with the CTAB plant genomic DNA isolation method from micro-propagated F1 hybrids and *J. microcarpa* and cv. Serr parents. DNA was diluted to the uniform concentration of 55 ng/µL. GBS involved complexity reduction by PstI restriction enzyme digestion. Groups of 96 samples including both parents were multiplexed using unique barcodes, and single-end sequenced with the Illumina HiSeq2000 platform at the Genomic Diversity Facility of Institute of Biotechnology, Cornell University. SNPs were called with Tassel-GBS pipeline v2.0 using default settings except that a minimum mapping quality of 2 was used in SAMToGBSdbPlugin. Illumina reads were deconvoluted using the barcodes, and reads were filtered and trimmed to 64 bp to construct unique tags. The *J. microcarpa* Jm31.01_v1.0 and *J. regia* JrSerr_v1.0 genome sequences were combined into a single fasta file and used as a reference. Unique tags were aligned onto the reference sequences with the BWA-aln aligner. High-quality SNPs were selected among SNPs obtained from the Tassel-GBS pipeline with VCFtools, BCFtools, and in-house awk and Python scripts. Non-segregating SNPs and calls below a threshold of five reads were removed.

The following convention was implemented in naming the markers. Each name was started with that of the mapping population followed by the abbreviated names of the genome and chromosome, and ended with the location of the SNP in the registry of the corresponding JrSerr_v1.0 or Jm31.01_v1.0 pseudomolecule” [6].

### 4.3. Phenotypic Analysis

The phenotyping of these hybrids for resistance to *A. tumefaciens* and *Phytophthora* spp. has been described in detail [6]. Briefly, the phenotype used for *A. tumefaciens* disease score was a visually assessed rank on a scale of one (no gall) to four (complete girdling of the plant from gall). The phenotype used for *Phytophthora* spp. disease score was a visually assessed percentage of crown or root rotted after pathogen inoculation of either *P. cinnamomi* or *P. pini*. The average percentages of crown and root rot for *P. cinnamomi* and *P. pini* were entered into the analysis and were then referred to as “*Phytophthora* spp.”.

To phenotype hybrids for *P. vulnus* counts, at least six clonal saplings of each hybrid and commercial comparatives RX1 and VX211 [23,24] were planted in a series of field experiments from 2014 to 2018. The hybrids used in this study were propagated from tissue cultures and developed into saplings in greenhouse culture. At the Kearney Agricultural Research and Extension Center (36.6015° N, 119.5109° W), saplings were planted in rows of 3.35 m distance at 1.65 m spacing within the row. About one month after planting, every tree was concomitantly inoculated with ~1000 vermiform *P. vulnus* and second-stage juveniles (J2) of *Meloidogyne incognita* by dispensing infested field soil from underneath infected perennial crops at the base of the tree. Selected trees of the genotype groups were chosen for root collections for nematode evaluations. A 20–25 cm-deep trench was dug next to the tree to collect young roots of the respective tree genotype avoiding suberized roots. Kept cool in plastic bags until processing within 48 h of collection, the roots were chopped into 1.2 cm pieces and 20 g portions placed on top of Baermann funnels. In a mist chamber apparatus, the roots were intermittently sprayed with water at 27 °C for five days. After this, the extracts were collected and *P. vulnus* were identified and counted [25]. Nematode numbers were recorded on a per 1 g basis. In each dormant season, the height of each of the trees in these nematode field screens was measured from the ground level perpendicularly to the maximum height of the trees with an extended ruler. Thus, the tree height was measured under nematode-infested conditions.

### 4.4. Data Cleaning and Preparation

The genotype data for 31.01 and 31.09 and cv. Serr “Serr” analysis were cleaned using the snpReady package in R [26]. The data used for QTL mapping and marker-assisted selection/genomic selection were converted from “a” to 1 and “b” to 2. Using the raw.data() function, SNP data from the genotype matrix were cleaned by removing markers with an MAF of 0.1, a sweep sample rate of 0.5 or higher, a call rate of 0.95 or higher, and imputing data points with the “k-nearest markers” method. See the raw.data() function from the snpReady package for more details. The data used for QTL mapping was not imputed and was converted back to a/b coding before analysis.

### 4.5. QTL Mapping

We performed QTL mapping using the “QTL” package in R [27]. After loading the data as a cross object, we calculated the genotype probabilities and then performed a single QTL scan using the marker regression (mr) method. To further refine the mapping step, we performed permutation testing via 1000 permutations to calculate a significance threshold at alpha = 0.05 for each marker’s Logarithm of the Odds (LOD) score. These LOD scores and significance thresholds were used to generate the LOD plots (Figure 1 and Figure 2). To estimate the total variation explained by each QTL, we built a multiple regression model in R with all significant markers within the QTL and reported the adjusted R2 of the full model. We performed the same analysis for the top three markers.

### 4.6. Prediction and Selection Performance Estimation

RF and rrBLUP approaches were used on each trait in this study to assess the markers’ predictive power. To assess model accuracy for RF and rrBLUP approaches while lowering the risk of overfitting, we used 70% of the data for training and 30% for testing.

After evaluating different numbers of top markers and their prediction accuracies with the *rfe* function from the caret package in R, we opted to utilize the top eight markers as described in the results. The rrBLUP package utilizes all markers for prediction in a ridge regression approach, with critical differences over standard regression or random forest models. Please see the package information for more details [28].

For the RF approach, a random forest model was built on the training data and its top eight markers were extracted by mean decrease in impurity (MDI). Then, these markers were used as predictors to train a new random forest model for predicting the phenotypes of the testing data.

Prediction accuracy was assessed by calculating the Pearson correlation coefficient of the predicted vs. actual values of the testing data with a one-tailed alternative hypothesis predicting whether the correlation coefficient would be positive rather than negative. Marker selection and accuracy estimation was repeated 15 independent times, and the average *p*-value of these tests was reported. For selection performance, we used the same 70/30 training and testing splits to produce the predicted values. The testing values used for prediction were then arranged by their accompanying predicted values, and the top 25% of that data were extracted and defined as the “selected values”. These selected values were then compared to a “no selection” scenario in which all the testing values were kept. We used a Student’s *t*-test with a one-tailed alternative hypothesis predicting that the predicted values would be less (if disease score was being analyzed) or more (if tree height was being analyzed). This was repeated 15 independent times, and the average *p*-value of these tests was reported (Figure 6 and Table 6).

### 4.7. R Code and Availability

The R statistical software R version 4.4.0 (2024-04-24 ucrt) was used in Rstudio version 2023.6.0.421 to facilitate all statistical analysis and visualization produced in this study [29,30]. The following R packages were used:

caret [31], data.table [32], dplyr [33], ggplot [34], ggprism [35], ggtext [36], kableExtra [37], knitr [38], OmicsAnalyst [39], openxlsx [40], qtl [27], randomForest [41], rmarkdown [42], rrBLUP [28], sjPlot [43], snpReady [26], stringr [44], tibble [45], tidytable [46], tidytext [47], tidyr [48], webshot2 [49].

A repository for the code used in this study can be found at [https://github.com/hsaxe/Walnut_Root_QTL] (accessed on 1 January 2025).

## 5. Conclusions

This study provided further insights into the genetics of walnut root diseases. The co-location of tree height and the root system disease traits of nematode count, *Phytophthora* disease score, and crown gall disease score to a single QTL is a novel finding worth further investigation. It begs the question of how a single QTL could regulate many disease-related traits and tree height. Moreover, we provided actionable knowledge by leveraging these QTL to predict these traits using just the SNPs. Such knowledge provides the potential to improve the breeding efficiency of walnut rootstocks. Furthermore, fine mapping of this region could yield further benefits to both outcomes of this study. Fine mapping also paves the way for an optimized MAS pipeline by narrowing down the best markers to use for advanced selection of future, unphenotyped progeny. Lastly, this GBS data contain other variation that is not explained by any of the traits reported here. Therefore, the GBS data can serve as a resource to be further mined for QTL for drought resistance, blackline, or other agronomic traits of interest in walnut roots.

## Figures and Tables

**Table 1:** All significant markers from QTL mapping on the 31.01 × cv. Serr progeny with LOD threshold α = 0.05. This defines the QTL. Length is QTL length in base pairs. Markers are the number of unique markers within the QTL.

Trait	Chromosome	Unique Markers	Length (bp)	Percent Variance Explained
Two-year *P. vulnus* Counts	4D	31	12,109,447	13.9%
Three-year *P. vulnus* Counts	4D	17	5,298,334	13.1%
Two-year Tree Height	4D	65	30,660,994	70.3%
Three-year Tree Height	4D	65	30,660,994	41.4%
Three-year Tree Height	6D	14	3,493,793	4.8%
*A. tumefaciens* Disease Score	4D	59	30,060,179	45.4%
*Phytophthora* spp. Disease Score	4D	38	21,190,695	21.0%
*Phytophthora* spp. Disease Score	6D	3	3,720,218	6.1%

**Table 2:** Top markers by LOD from QTL mapping on the 31.01 × cv. Serr progeny.

Trait	Marker(s)	Chromosome	LOD	Length (bp)	Percent Variance Explained
Two-year *P. vulnus* Counts	31.01_Jm4D_26669075	4D	9.8	0	15.7%
Three-year *P. vulnus* Counts	31.01_Jm4D_26669075	4D	7.6	0	12.9%
Two-year Tree Height	31.01_Jm4D_26669075	4D	44.9	0	67.1%
Three-year Tree Height	31.01_Jm4D_26669075	4D	22.2	0	33.8%
*A. tumefaciens* Disease Score	31.01_Jm4D_26168643	4D	28.4	500,432	44.1%
*A. tumefaciens* Disease Score	31.01_Jm4D_26359154	4D	28.4	500,432	44.1%
*A. tumefaciens* Disease Score	31.01_Jm4D_26669075	4D	28.4	500,432	44.1%
*Phytophthora* spp. Disease Score	31.01_Jm4D_26168643	4D	11.1	500,432	18.9%
*Phytophthora* spp. Disease Score	31.01_Jm4D_26359154	4D	11.1	500,432	18.9%
*Phytophthora* spp. Disease Score	31.01_Jm4D_26669075	4D	11.1	500,432	18.9%

**Table 3:** All significant markers from QTL mapping on the 31.09 × cv. Serr progeny with LOD threshold α = 0.05.

Trait	Chromosome	Unique Markers	Length (bp)	Percent Variance Explained
Two-year *P. vulnus* Counts	3D	2	372,371	7.0%
Two-year *P. vulnus* Counts	4D	3	54,916	8.2%
Three-year *P. vulnus* Counts	1D	39	24,916,299	8.5%
Three-year *P. vulnus* Counts	4D	14	9,378,085	16.7%
Two-year Tree Height	4D	31	21,274,993	20.1%
Three-year Tree Height	4D	19	10,480,961	17.2%
*A. tumefaciens* Disease Score	4D	59	31,696,222	39.7%
*A. tumefaciens* Disease Score	7D	1	0	7.0%
*Phytophthora* spp. Disease Score	4D	46	29,549,284	17.5%

**Table 4:** Top markers by LOD from QTL mapping on the 31.09 × cv. Serr progeny.

Trait	Marker(s)	Chromosome	LOD	Length (bp)	Percent Variance Explained
Two-year *P. vulnus* Counts	31.09_Jm4D_23906200	4D	3.9	54,916	8.2%
Two-year *P. vulnus* Counts	31.09_Jm4D_23960954	4D	3.9	54,916	8.2%
Two-year *P. vulnus* Counts	31.09_Jm4D_23961116	4D	3.9	54,916	8.2%
Three-year *P. vulnus* Counts	31.09_Jm4D_26359154	4D	7.1	0	17.3%
Two-year Tree Height	31.09_Jm4D_25101968	4D	6.2	0	23.5%
Three-year Tree Height	31.09_Jm4D_26359154	4D	7.5	0	17.1%
*A. tumefaciens* Disease Score	31.09_Jm4D_26359154	4D	18.9	0	36.9%
*Phytophthora* spp. Disease Score	31.09_Jm4D_23816262	4D	11.0	443,002	17.6%
*Phytophthora* spp. Disease Score	31.09_Jm4D_24259264	4D	11.0	443,002	17.6%

**Table 5:** Prediction accuracies (r) and their significance for each trait assessed by Pearson correlation of model-predicted vs. actual values. Each value in the table is the average correlation coefficient representing prediction accuracy estimates. Stars indicate the average *p*-value for each boxplot of correlations. “***” average *p*-value less than 0.001, “**” average *p*-value between 0.01 and 0.001, “*” average *p*-value between 0.05 and 0.01.

Method	Female Parent	*A. tumefaciens* Disease Score	*Phytophthora* spp. Disease Score	Three-Year Tree Height	Three-Year *P. vulnus* Counts	Two-Year Tree Height	Two-Year *P. vulnus* Counts
RF	31.01	0.67 ***	0.49 **	0.59 ***	0.33 **	0.84 ***	0.35 **
RF	31.09	0.58 ***	0.38 **	0.31 *	0.34 *	0.46 *	0.19
rrBLUP	31.01	0.46 ***	0.36 **	0.52 ***	0.24	0.73 ***	0.25 *
rrBLUP	31.09	0.42 **	0.25 *	0.37 *	0.31 *	0.51 *	0.19

**Table 6:** Selection performance by trait, method, and female parent. Each cell represents average phenotypic values of traits comparing *t*-test estimates of using selection vs. no. Stars indicate the average *p*-value for each boxplot of correlations. “***” average *p*-value less than 0.001, “**” average *p*-value between 0.01 and 0.001, “*” average *p*-value between 0.05 and 0.01, “.” Average *p*-value between 0.01 and 0.1.

Stat	Method	Female Parent	*A. tumefaciens* Disease Score	*Phytophthora* spp. Disease Score	Three-Year Tree Height	Three-Year *P. vulnus* Counts	Two-Year Tree Height	Two-Year *P. vulnus* Counts
Selection	RF	31.01	1.54 ***	25.8 *	134 .	30.1 *	127 ***	5.02 .
No Selection	RF	31.01	2.36 ***	37.9 *	112 .	59.3 *	89.3 ***	12.5 .
Selection	RF	31.09	1.5 ***	16.5 *	113	46.7 .	98.4 *	13.9
No Selection	RF	31.09	2.32 ***	26.4 *	99.3	94.1 .	70.6 *	19.4
Selection	rrBLUP	31.01	1.72 **	31	143 *	40.3	124 ***	6.47
No Selection	rrBLUP	31.01	2.36 **	37.9	112 *	59.3	89.3 ***	12.8
Selection	rrBLUP	31.09	1.84 .	19.9 .	120 .	54.4	86.6	13.5
No Selection	rrBLUP	31.09	2.32 .	26.4 .	99.3 .	94.1	70.6	19.6

## Data Availability

Genotype data were obtained by downloading from Supplementary Table S2 of [6]. The phenotypic data used in this study are available as Supplementary Data (Files S5–S7).

## Supplementary Materials

The following supporting information can be downloaded at: https://www.mdpi.com/article/10.3390/ijms26030903/s1.
