# Supplementary figures and images for: Co-Location of QTL for Vigor and Resistance to Three Diseases in Juglans microcarpa × J. regia Rootstocks
Source: Int J Mol Sci. 2025 Jan 22;26(3):903. doi: 10.3390/ijms26030903 (PMC11817649; doi:10.3390/ijms26030903)

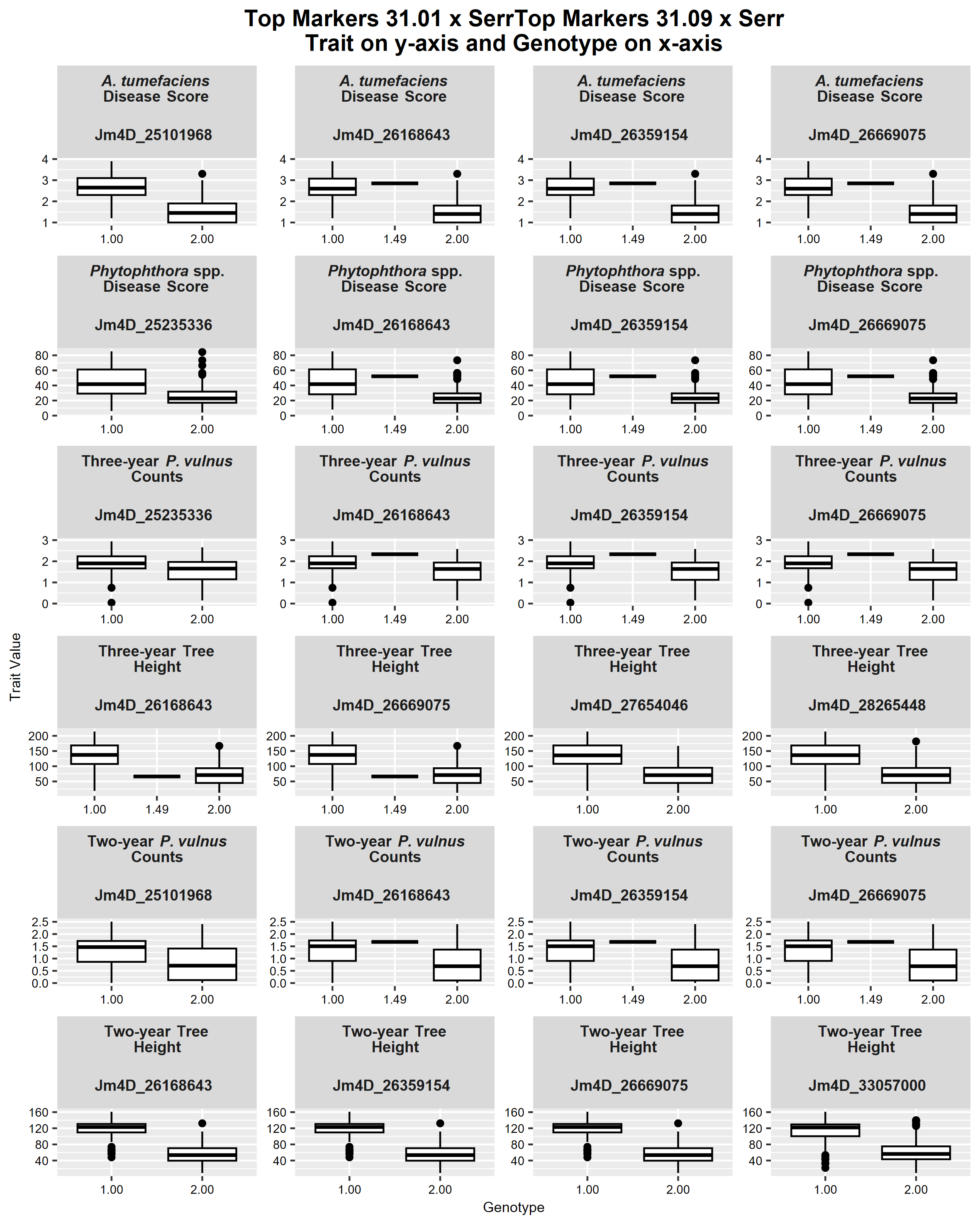

Supplement: Supplementary file 1 [file ijms-26-00903-s001.zip › ijms-3366761-SI/Final_Figs/Figure_S1.png]

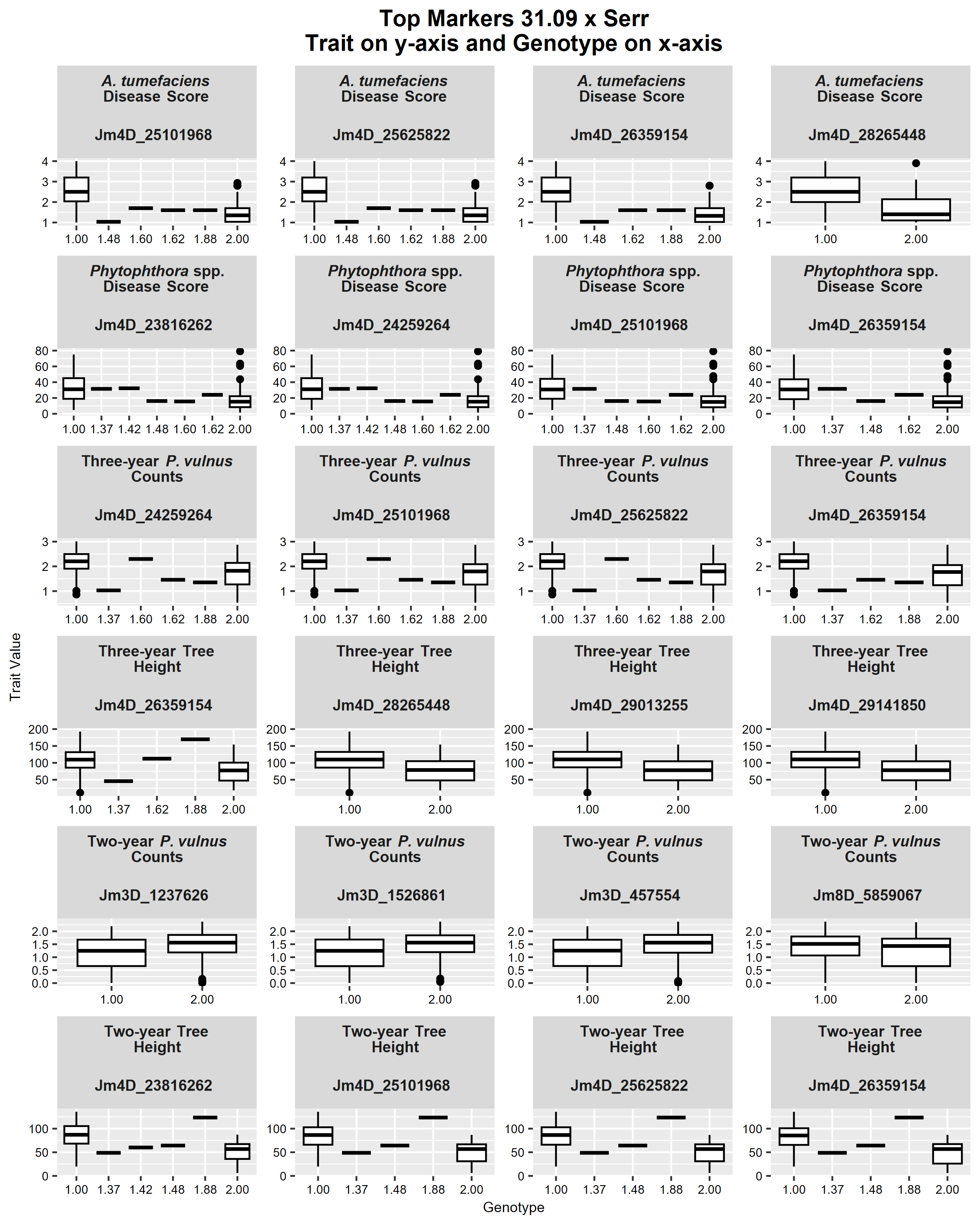

Supplement: Supplementary file 1 [file ijms-26-00903-s001.zip › ijms-3366761-SI/Final_Figs/Figure_S2.png]
